# Egg Phospholipids and Cardiovascular Health

**DOI:** 10.3390/nu7042731

**Published:** 2015-04-13

**Authors:** Christopher N. Blesso

**Affiliations:** Department of Nutritional Sciences, University of Connecticut, Storrs, CT 06269, USA; E-Mail: christopher.blesso@uconn.edu; Tel.: +860-486-9049; Fax: +860-486-3674

**Keywords:** atherosclerosis, cardiovascular disease, egg, HDL, phosphatidylcholine, phospholipids, sphingomyelin, TMAO

## Abstract

Eggs are a major source of phospholipids (PL) in the Western diet. Dietary PL have emerged as a potential source of bioactive lipids that may have widespread effects on pathways related to inflammation, cholesterol metabolism, and high-density lipoprotein (HDL) function. Based on pre-clinical studies, egg phosphatidylcholine (PC) and sphingomyelin appear to regulate cholesterol absorption and inflammation. In clinical studies, egg PL intake is associated with beneficial changes in biomarkers related to HDL reverse cholesterol transport. Recently, egg PC was shown to be a substrate for the generation of trimethylamine *N*-oxide (TMAO), a gut microbe-dependent metabolite associated with increased cardiovascular disease (CVD) risk. More research is warranted to examine potential serum TMAO responses with chronic egg ingestion and in different populations, such as diabetics. In this review, the recent basic science, clinical, and epidemiological findings examining egg PL intake and risk of CVD are summarized.

## 1. Introduction

Cardiovascular disease (CVD) claims upwards of 17 million lives worldwide each year [1]. In the United States, more than one third of adults suffer from some form of CVD which accounts for approximately one out of three deaths [2]. Atherosclerosis is a key contributor to CVD and is characterized by the hardening and thickening of the artery wall caused by accumulation of fatty plaque. Atherosclerosis is an insidious and progressive chronic inflammatory disease that takes decades to develop in humans [3]. Atherosclerosis is not only an inflammatory disease characterized by infiltration of immune cells, but also a lipid disorder; subendothelial accumulation of lipids derived from plasma lipoproteins is a key initiator of plaque development [4]. Lipoprotein metabolism is therefore critical to the development of atherosclerosis [5]. Lipoproteins have evolved to facilitate the extracellular transport of water-insoluble lipids in multicellular organisms [6]. Apolipoprotein B-containing lipoproteins that originate from the liver, such as very-low-density lipoprotein (VLDL) and low-density lipoprotein (LDL), contribute to the CVD process. In contrast, high-density lipoprotein (HDL) improves CVD through its ability to remove excess lipid from the artery and transport it back to the liver for excretion from the body, a pathway termed “reverse cholesterol transport” (RCT) [7]. The atheroprotective effect of HDL is mainly attributed to its role in RCT, with plasma HDL-cholesterol (HDL-C) considered to be a surrogate metric for this pathway [8]. The relationship between blood cholesterol and CVD is well-established, with the lowering of LDL-cholesterol (LDL-C) levels being the primary target of preventive therapy [9]. There has also been considerable interest in studying the relationship between dietary cholesterol intake and CVD risk [10]. Eggs are one of the richest sources of cholesterol in the diet. However, numerous large-scale epidemiological studies have failed to find any association between the intake of eggs and CVD risk [11,12,13]. This lack of association may be related to the other factors found in eggs that may influence CVD risk, such as the antioxidant carotenoids lutein and zeaxanthin [14]. Besides being an important contributor of dietary cholesterol in the Western diet, eggs are also a rich source of phospholipids (PL) [15]. Dietary PL have emerged as a potential source of bioactive lipids that may have widespread effects on pathways related to inflammation, cholesterol metabolism, and HDL function. The aim of this review is to summarize the recent basic science, clinical, and epidemiological research examining egg PL intake and CVD risk.

## 2. Phospholipid Content and Composition of the Chicken Egg

**Figure 1:**
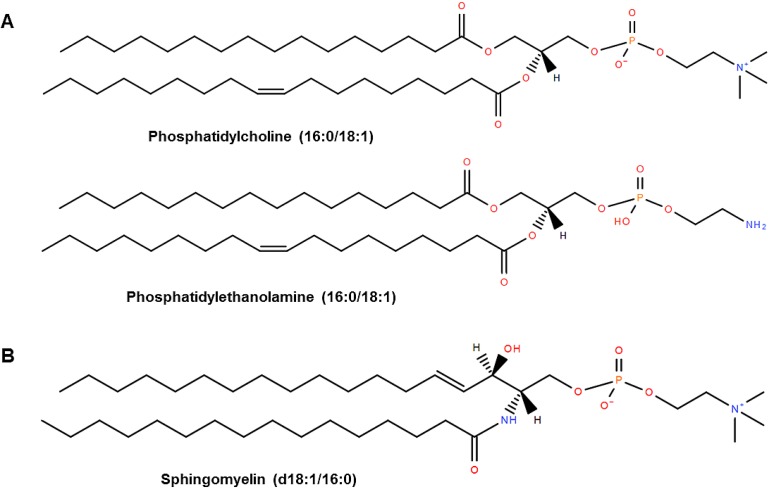
Structures of major phospholipids in egg yolk. Major molecular species of egg glycerophospholipids (**A**) and sphingomyelin (**B**). Lipid structures were drawn using Lipid MAPS tools [31].

PL are key components of all biological membranes and are abundantly found in the diet, primarily as glycerophospholipid and sphingolipid classes. Dietary glycerophospholipids are made up of two fatty acids (FA), a glycerol backbone, a phosphate group, and a polar organic molecule (choline, serine, inositol, or ethanolamine) (Figure 1A). Dietary glycerophospholipids are primarily absorbed in the gastrointestinal (GI) tract as lysophospholipids and free FA after pancreatic phospholipase A_2_ (PLA_2_) hydrolyzes the fatty acyl bond at the *sn*-2 position [16]. Glycerophospholipids are absorbed into the GI tract with high efficiency, for example, >90% of phosphatidylcholine (PC) is absorbed [17]. Dietary sphingolipids are primarily in the form of sphingomyelin (SM) [18], which consists of a ceramide (a FA linked to a long-chain sphingoid base through an amide linkage) with a phosphorylcholine head group (Figure 1B). Digestion of SM in the intestine is slow and incomplete, with initial hydrolysis of SM to ceramide by alkaline sphingomyelinase and subsequent hydrolysis to sphingosine by neutral ceramidase [19]. Both ceramide and sphingosine can be absorbed into intestinal mucosal cells [19]. Chicken eggs contain approximately 28% of total lipids by weight as PL, with the remaining 66% as triglycerides (TG) and 5% as cholesterol [20]. The average large egg contains approximately 1.3 g of PL [15,21], which are almost exclusively found in the yolk. A typical Western diet contains about 2–8 g of dietary PL per day [22]. Estimates of average egg intake in the U.S. [23] indicate that egg-derived PL contributes 10%–40% (or 0.8 g) of daily consumed PL. The major PL species found in egg include PC, phosphatidylethanolamine (PE), SM, and phosphatidylinositol (PI) [24]. The typical PL composition of egg is shown in Table 1, which reveals PC as the predominant species making up almost three quarters of the total PL. The typical FA compositions of egg PL species vary [25,26] and are shown in Table 1. These FA compositions can be influenced somewhat by modifying the dietary FA intake of the hen [25,27,28]. Egg PC typically consists primarily of palmitic acid (16:0) and oleic acid (18:1) at the *sn*-1 and *sn*-2 positions, respectively. The major PC molecular species include PC (16:0/18:1), PC (22:6/16:0), and PC (22:6/16:1) [28,29]. Egg PE consists primarily of saturated FA such as stearic acid (18:0) at the *sn*-1 position, with a balanced mixture of unsaturated FA at the *sn*-2 position. The major PE molecular species include PE (16:0/18:1), PE (18:0/18:1), PE (18:0/18:2), and PE 18:0/20:4 [28,29,30]. Egg SM contains primarily saturated FA with palmitic acid (16:0) and stearic acid (18:0) making up ~80% of SM FA.

## 3. Egg Phospholipids and Lipid Absorption

Inhibition of luminal cholesterol absorption is an attractive target to lower blood cholesterol and reduce CVD risk. Cholesterol absorption is widely recognized to influence serum lipids [32]. Pharmacological agents, such as ezetimibe, have been developed to reduce intestinal cholesterol absorption as a means of lowering serum cholesterol and CVD risk [33]. Large intakes of dietary lecithin have long been known to influence serum cholesterol levels in humans [34]. Meta-analysis of soy lecithin trials has suggested that the unsaturated FA component was primarily responsible for the hypocholesterolemic effects observed in early studies [35]. However, recent studies with more saturated sources of PL, such as egg, show that intact PL strongly influence lipid absorption through molecular interactions. Dietary phospholipids are known to inhibit cholesterol absorption when added in significant amounts to the diet (as reviewed by Cohn *et al.* [22]). Animal studies have shown that egg PL, such as PC and SM, reduce cholesterol and FA absorption by possibly interfering with lipid mobilization from mixed micelles [36,37,38,39].

**Table 1:** Typical Composition of Egg Phospholipids.

**Egg PL Composition [24]**									
Egg Yolk	PC	PE	LysoPC	SM	LysoPE	PI			
Concentration (mg/100 g yolk)	5840	1500	270	190	90	330			
Percentage of total PL (%)	71.1	18.3	3.3	2.3	1.1	4.0			
**Egg PC FA Composition [25]**									
Position	16:0	16:1	18:0	18:1	18:2	20:4	22:6		
*sn*-1	62.8	1.4	27.2	6.5	0.8	Trace	-		
*sn*-2	6	1	2	56	24.5	5.6	2.9		
Total	31	1.2	14.1	33.4	13.4	3.3	1.9		
**Egg PE FA Composition [25]**									
Position	16:0	18:0	18:1	18:2	20:4	22:6			
*sn*-1	34.2	51.2	10.5	0.3	Trace	-			
*sn*-2	7	4.3	32.1	17.5	23.9	11			
Total	16.4	28.3	21.5	10.2	14	6.8			
**Egg SM FA Composition Adapted from [26]**									
16:0	18:0	18:1	20:0	22:0	22:1	23:0	23:1	24:0	24:1
68	10	1	4	6	1	2	-	3	6

Abbreviations: FA, fatty acid; LysoPC, lysophosphatidylcholine; LysoPE, lysophosphatidylethanolamine; PC, phosphatidylcholine; PE, phosphatidylethanolamine; PI, phosphatidylinositol; PL, phospholipid; SM, sphingomyelin.

Although biliary PC is a critical emulsifier of dietary lipids and aids in their digestion and absorption in the GI tract, excess luminal PC appears to inhibit lipid absorption. Young and Hui [40] showed that hydrolysis of surface PC by pancreatic PLA_2_ is required for proper pancreatic lipase/colipase digestion of TG and absorption of cholesterol and FA from lipid emulsions. With high PL concentrations in lipid emulsions (>0.3 PL/TG molar ratio), pancreatic lipase/colipase hydrolysis of TG was impaired and cholesterol absorption into rat IEC-6 intestinal cells was diminished [40]. Furthermore, micellar PC was shown to inhibit dietary cholesterol absorption into Caco-2 cells, which was reversed by conversion to lysophosphatidylcholine by pancreatic PLA_2_ [41]. PC-enriched micelles appear to impede diffusion across the unstirred water layer of the intestine [42,43]. Thus, digestion of surface PC appears to be necessary for proper absorption of lipids from both lipid emulsions and micelles. Jiang *et al.* [36] showed that duodenal infusion of a lipid emulsion containing egg PC significantly reduced cholesterol absorption by ~20% in lymph duct cannulated rats. The influence of PC on cholesterol absorption appears to be dependent on FA saturation, as egg PC inhibited the absorption of cholesterol into lymphatics greater than the more unsaturated soy PC in the same study. Soy PC infusion actually increased cholesterol and FA absorption compared to no PC control lipid emulsion. Furthermore, hydrogenated egg PC had a greater effect on cholesterol and TG absorption than egg PC. PC that is saturated at the *sn*-1 position is known to be a poor substrate for pancreatic PLA_2_ hydrolysis [44]. Thus, it appears that the saturation of egg PC makes it more effective at inhibiting cholesterol absorption than more unsaturated PC, such as soy PC.

SM and other sphingolipids have been shown to dose-dependently reduce the absorption of cholesterol, TG, and FA in rodents [37,38,39]. SM interacts with cholesterol with high affinity and appears to alter its micellar solubilization. The amide portion of SM can interact with the hydroxyl group of cholesterol through hydrogen bonding [45]. Furthermore, the strength of association appears to be influenced by SM FA chain length and saturation [38]. Ceramide and sphingosine, which are products of SM digestion, also appear to reduce lipid absorption into intestinal cells through hydrogen bonding with the hydroxyl group of cholesterol [46] and possibly through interactions with the carboxylic acid group of FA [39]. Sphingosine can form complexes with cholesterol and limit uptake via the cholesterol transporter Niemann-Pick C1 like 1 (NPC1L1) [46]. Duivenvoorden *et al.* [39] supplemented the diets of APOE*3Leiden mice with different types of sphingolipids (including SM, ceramide, and sphingosine) and examined their effects on plasma lipids. Dietary sphingolipids dose-dependently lowered plasma cholesterol and TG in Western-type diet-fed mice through an inhibition of luminal FA and cholesterol absorption. While egg SM makes up only about 2% of total PL in egg yolk [24], this amount may still influence cholesterol absorption. Feeding of 0.2% and 0.4% egg SM to Western-type diet-fed APOE*3Leiden mice resulted in plasma cholesterol reductions of >20% [39]. Dried egg powder contains about 0.25% SM by weight, so this could potentially have an impact on inhibiting blood cholesterol changes that would normally occur from ingesting the amount of cholesterol found in the yolk. Milk SM has been shown to be a more potent inhibitor of cholesterol and FA absorption when compared to egg SM [38]. The greater inhibitory effect of milk SM on lipid absorption appears to be associated with its greater saturation and longer chain-length of its fatty acyl group, which may allow for stronger hydrophobic interactions [38]. Milk SM primarily consists of very long-chain FA (22:0, 23:0, 24:0), whereas egg SM consists primarily of the long-chain FA palmitic acid (16:0) [38].

Feeding of dietary PE has been shown to lower serum cholesterol in rats [47,48]. Mono- and di-unsaturated PE may influence cholesterol absorption like SM, as it has been shown to display a similar affinity to cholesterol as SM [49,50]. Both 1-palmitoyl-2-oleoyl-*sn*-glycero-3-phosphoethanolamine (POPE) [49] and 1-stearoyl-2-linoleoyl-*sn*-glycero-3-phosphoethanolamine (SLPE) [50], major PE molecular species in egg yolk [30], have been shown to interact with cholesterol in monolayers to a similar degree as SM; this suggests an important role for PE in lipid raft formation at the inner membrane leaflet of cells where it is most abundant. Due to the high affinity for cholesterol observed with certain PE species, dietary PE from egg may influence the absorption of luminal cholesterol similar to dietary SM.

## 4. Egg Phospholipids and Hepatic Lipid Metabolism

In animal models, egg PL appear to influence hepatic lipid metabolism through effects on cholesterol and bile acid synthesis, FA oxidation, and lipoprotein secretion [51,52,53]. Hepatic lipid levels are often shown to be reduced by dietary PL in animals, and this may be due to indirect effects via inhibition of intestinal lipid absorption and direct effects on hepatic nuclear receptors that regulate lipid metabolism. Feeding rats an egg yolk-enriched diet (~5% egg PL by weight) for 12 weeks lowered serum and hepatic lipids, and increased fecal sterol excretion compared to a cholesterol and fat-matched control group [51]. These changes were related to lower hepatic expression of cholesterol biosynthesis genes and increased expression of bile acid synthesis genes.

Peroxisome proliferator-activated receptor-α (PPARα) is a nuclear receptor abundantly expressed in the liver, where it functions as a key regulator of hepatic FA metabolism through transcriptional control of beta oxidation-related genes [52]. *In vitro* and rodent studies suggest that PC, specifically the 16:0 and 18:1-containing species PC (16:0/18:1), is a natural agonist of hepatic PPARα. Chakravarthy *et al.* [52] showed that portal vein infusion of PC (16:0/18:1) reduced hepatic steatosis in C57BL/6 mice and induced PPARα-dependent gene expression; although, this effect was not observed with intraperitoneal administration. Furthermore, incubation of PC (16:0/18:1) with the Hepa 1–6 mouse hepatoma cell line induced PPARα-target genes involved in FA oxidation. PC (16:0/18:1) was found to be a minor PL species in the liver which suggests it acts as a signaling molecule in this organ. Thus, this data suggests that providing PC (16:0/18:1) in the diet may affect hepatic PPARα activation if sufficient amounts reach the liver, as this is only a minor PL species in this organ [52]. Interestingly, PC (16:0/18:1) is the major PC species in eggs [29]. However, Cohn and colleagues [54] showed that feeding C57BL/6 mice a high fat diet supplemented with egg PC (1.25% by weight) for 3 weeks did not alter hepatic lipids and expression of a PPARα target gene (acyl-CoA oxidase) compared to control high fat diet. In contrast, feeding mice a hydrogenated form of egg PC, which contained only saturated FA, significantly lowered hepatic lipid levels [54]. Hydrogenated egg PC is associated with a greater inhibition of lipid absorption in rodents compared to natural egg PC [36]. Thus, it appears that at least in mice, the effects of short-term feeding of PC on hepatic lipids may related to its interference with lipid absorption, rather than modulation of PPARα.

Rye and colleagues [53] studied the effects of egg SM on hepatic lipid metabolism in Western-type diet-fed C57BL/6 mice. Egg SM feeding (0.6% of diet by weight) for 18 days was shown to reduce hepatic lipid levels and increase fecal cholesterol output compared to control mice. Although egg SM feeding substantially reduced hepatic lipids and led to a ~30% reduction in cholesterol absorption, it did not affect plasma cholesterol or TG levels. Sphingoid bases have been shown to activate PPARα transcriptional activity *in vitro* [55]. However, egg SM feeding of Western-type diet-fed mice reduced the hepatic expression of PPARα-target genes involved in FA oxidation [53]. The reduced hepatic lipid levels in egg SM-fed mice coincided with significantly lower hepatic expression of genes involved in cholesterol and FA metabolism [53].

The choline moiety of PL also appears to influence hepatic lipid metabolism and lipoprotein production. PC and SM are both sources of choline in the Western diet. Choline is an essential nutrient and is not synthesized in adequate amounts to meet the needs of humans [56]. Dietary choline is considered especially important for maintaining a healthy liver [57]. Methionine- and choline-deficient diets (MCD) are well-known inducers of liver injury in mice and are used as a model of nonalcoholic steatohepatitis [58]. This combined nutrient deficiency causes hepatic lipid accumulation by enhancing lipid uptake and reducing VLDL secretion [58]. In humans, a single-nucleotide polymorphism in the PC-synthesizing enzyme, phosphatidylethanolamine *N*-methyltransferase (PEMT), is associated with greater risk for non-alcoholic fatty liver disease (NAFLD) [59]. Dietary choline may also influence more advanced stages of liver disease, as low intake of choline was shown to be associated with increased hepatic fibrosis in postmenopausal women with NAFLD [60].

While there has been no reports specifically examining the effects of dietary egg PE on hepatic lipid metabolism, feeding soy PE to rats has been shown to lower hepatic cholesterol levels [47]. Soy PE consists of primarily PE (16:0/18:2), PE (18:2/18:2), and PE (16:0/18:1) molecular species [30].

## 5. Egg Phospholipids and HDL Metabolism

Therapies aimed at increasing plasma HDL-C may be beneficial for preventing CVD. Plasma HDL-C has been shown to be inversely related to the extent of atherosclerosis [61]. According to data from large prospective cohort studies, it can be estimated that for every 1 mg/dL (0.0259 mmol/L) increase in HDL-C, there is an approximate 2–3% reduction in CVD risk [62]. Strong experimental evidence confirms that atherosclerosis is directly alleviated by HDL [63,64,65]. HDL is thought to improve CVD outcomes largely by its ability to remove cholesterol from the artery via RCT. Regular egg yolk consumption has been shown to increase plasma HDL-C and increase the mean size of HDL particles in healthy [66], overweight [67], and metabolic syndrome (MetS) populations [68]. These increases in HDL-C and HDL size may be due to the high intake of egg PL.

PL feeding has been associated with increases in HDL-C in animal and human studies [69,70]. PL content of HDL has been shown to be a major factor in its ability to accept cholesterol from cells during the initial stages of RCT [71,72]. Dietary PC has been observed to preferentially incorporate into HDL particles after ingestion. Zierenberg and Grundy [17] examined the metabolic fate of ^3^H/^14^C-labeled polyunsaturated PC in men. Soybean PC was labeled in its FA (^14^C) and choline moiety (^3^H), given orally at a dose of 1 g, and then blood was collected from 6–24 h post-ingestion. The ^4^C and ^3^H radiolabels preferentially incorporated into plasma lipoproteins compared to red blood cells, with higher specific activity appearing in plasma HDL than in apoB-containing lipoproteins. This increased appearance of radiolabeled PC in plasma HDL may be explained by the exchange of chylomicron-PC to HDL in circulation or direct secretion of PC into nascent intestinally-derived HDL. Tall *et al.* [73] examined the incorporation of oral ^3^H/^14^C-labeled polyunsaturated PC into HDL subclasses. Major peaks in radiolabel specific activity appeared after 5–8 h in the PL fraction of several HDL subclasses, in the order of HDL_2a_ (1.11–1.12 g/mL) > HDL_3_ (1.19 g/mL) > HDL_2b_ (1.07–1.09 g/mL). Thus, the results suggest that the HDL_2a_ subclass is the major HDL acceptor of oral PL, but all HDL subclasses appear to incorporate dietary PL to some extent. Plasma HDL-PL content has been shown to increase postprandially after egg yolk feeding [74]. Thus, dietary egg PL appears in serum HDL after meals and may impact HDL function.

Clinical studies where subjects were fed egg yolks demonstrated improvements in other markers of RCT besides HDL-C, such as plasma lecithin-cholesterol acyltransferase (LCAT) activity [68,75,76] and serum cholesterol efflux capacity [77]. Recently, HDL cholesterol efflux capacity has emerged as a significant predictor of heart disease status, even after adjusting for plasma HDL-C and its major protein, apolipoprotein (apo) A-I [78]. PL-enrichment of HDL with either PC or SM improves its ability to mobilize cholesterol from cells [71,72]. The first component of RCT, involving cellular cholesterol mobilization, relies on apo A-I and HDL particle interactions to promote cholesterol efflux by a variety of passive and active mechanisms [79]. Important factors in HDL cellular cholesterol efflux include cholesterol mobilization via ABC transporters (ATP-binding cassette transporters A1 and G1) and scavenger receptor B1 (SR-B1), desorption of free cholesterol via aqueous diffusion, and esterification of cholesterol by LCAT [79]. Preferred acceptors for ABCG1 and SR-B1 are larger HDL particles containing PL [71,72]. Egg yolk feeding was shown to increase HDL-PE and decrease HDL-TG [77]. Interestingly, egg ingestion shifted HDL-SM towards molecular species that more closely resembled egg SM, suggesting that egg SM may be incorporated into HDL [77]. Thus, increasing dietary intake of egg PL may change HDL-PL content and this could explain the increases in serum cholesterol efflux capacity and enlargement in HDL particle size observed with egg yolk intake in humans [68,77]. The increases in HDL-C associated with egg PL intake may reflect a greater capacity for RCT.

## 6. Egg Phospholipids and Inflammation

In addition to effects on lipid metabolism, dietary intake of egg PL may also reduce inflammation. Consuming three eggs per day for 12 weeks resulted in a reduction in plasma C-reactive protein (CRP) and an increase in adiponectin in overweight men; changes which were not observed with yolk-free egg substitute [80]. Egg consumption has also led to improvements in circulating plasma inflammatory markers in adults with MetS [81]. In combination with moderate carbohydrate restriction, the addition of three eggs per day led to decreases in plasma TNFα and serum amyloid A in men and women with MetS, whereas no changes were observed in subjects consuming a yolk-free egg substitute [81]. Egg yolk contains a significant amount of choline as PC. Dietary choline intake has been shown to be inversely associated with serum inflammation markers in healthy adults [82]. Choline administration to mice has been shown to significantly reduce plasma TNFα and enhance survival in response to an endotoxin challenge [83]. Choline seems to act through nicotinic acetylcholine receptor subunit α7 (α7nAChR) activation, which has a tonic inhibitory effect on immune cell inflammatory responses [83,84]. Some studies have suggested that dietary PC may reduce inflammation in the GI tract [85,86]. Therapeutic usage of oral PC (>1 g daily) in ulcerative colitis is well-documented and improves inflammation [85,86]. This GI anti-inflammatory effect of PC may be of significance to MetS, as there is evidence linking GI inflammation to the development of obesity and insulin resistance in animal models [87]. Furthermore, intestinal inflammation is associated with impaired formation of intestinally-derived nascent HDL in Crohn’s disease [88,89].

## 7. Egg Phospholipids and Trimethylamine *N*-Oxide (TMAO) Formation

Recently, Hazen and colleagues [90] used unbiased metabolomics to identify three metabolites of dietary PC (choline, TMAO, and betaine) which were predictors of CVD in a large clinical cohort. The presumed atherogenic factor, TMAO, is a known metabolite of dietary choline. Dietary choline can undergo catabolism in the GI tract by gut microflora to form the gas trimethylamine (TMA), which is absorbed and rapidly oxidized to TMAO in the liver by the hepatic enzyme flavin containing monooxygenase 3 (FMO3) [91,92]. Hazen and colleagues [90] showed that feeding apoE^−/−^ mice diets rich in either choline (0.5% or 1% wt/wt) or TMAO (0.12% wt/wt) for 20 weeks resulted in increased aortic root lesion size despite no changes in traditional CVD risk biomarkers. Highlighting the role of gut microflora in this process, TMAO formation from dietary PC and the atherogenic effect of choline feeding were abolished when the mice were germ-free or co-administered broad spectrum antibiotics to deplete gut bacteria. Additionally, the plasma levels of TMAO in mice and humans were associated with the expression of hepatic FMO3. Thus, formation of blood TMAO subsequent to dietary PC intake requires intact gut microflora and may be influenced by hepatic FMO3 expression. Interestingly, TMAO levels do not appear to be strongly influenced by genetic factors in humans [93], suggesting that diet and gut microflora primarily determine differences in plasma TMAO levels between individuals. TMAO formation was also shown to be formed from the consumption of another dietary trimethylamine compound, carnitine [94]. Subsequently, the relationship between TMAO and CVD was confirmed in a larger prospective cohort [95]. The investigators suggested that the atherogenic effect of TMAO is due to inhibition of RCT and increased macrophage scavenger receptor expression, resulting in enhanced foam cell formation [96]. Increases in plasma TMAO that occurred after carnitine and choline feeding of mice were associated with impaired RCT at the stage of bile acid production [94]. TMAO was also shown to induce the scavenger receptors CD36 and SR-A in macrophages [90,94].

Notably, it was shown that healthy participants who consumed a dietary PC challenge (hard-boiled eggs and deuterium (d9)-labeled PC) had acute increases in plasma TMAO in a gut microflora-dependent manner [95]. Furthermore, Zeisel and colleagues [97] performed a small, double-blind, randomized dose-response study where healthy subjects (*n* = 6) consumed a breakfast with 0, 1, 2, 4 or 6 egg yolks and levels of plasma TMAO were measured at pre- and post-breakfast time intervals (up to 24 h). Egg intake dose-dependently increased mean plasma TMAO area under the curve (AUC) values, with TMAO response peaks occurring 6–8 h after ingestion. Interestingly, there were >4-fold differences in plasma TMAO levels between subjects at similar dosages, potentially due to differences in gut microflora or hepatic FMO3 activity. Regardless of the large interindividual variation in plasma TMAO, each subject displayed an increase in plasma TMAO when increasing the number of egg yolks consumed. Collectively, these two studies demonstrate that acute intake of PC from egg may influence postprandial plasma TMAO levels in humans. The effect of chronic egg intake on TMAO levels has not been examined yet. Findings from chronic egg intake studies would be more meaningful than acute egg feedings in regards to atherosclerosis risk, since it is a disease that takes decades to develop. Chronic egg intake could influence gut microflora composition and this would presumably impact TMAO formation from choline, such as that observed with chronic exposure to carnitine [94]. It remains to be seen whether there is an “optimal” egg dose, where the RCT-promoting aspects or other HDL-promoting effects outweigh any possible TMAO effects on RCT and CVD risk. It will also be important to determine whether chronic or acute egg intake in humans affects the expression of scavenger receptors in peripheral blood mononuclear cells (PBMCs). Although egg ingestion may increase plasma TMAO to some extent, it potentially may not reach the threshold level needed to affect scavenger receptor expression.

The majority of luminal PC available for absorption comes from the endogenously-derived PL found in bile (10–20 g/day) [98]. Biliary PC, however, does not seem to be a major contributor to TMAO levels, as plasma TMAO is low in the absence of large amounts of dietary choline [90]. It is unclear why the large amounts of biliary PC normally found in the intestine do not result in very high levels of plasma TMAO regardless of dietary PC intake. High amounts of dietary PC, particularly saturated PC [44], may overwhelm pancreatic PLA_2_ activity since it is known to be less efficient than other lipases [16]. Intact PC digestion in PLA_2_^−/−^ mice demonstrates additional enzymes in the intestine are capable of phospholipase activity [99]. Ileal phospholipase B acts in the distal intestinal mucosa to hydrolyze PC [100]. Thus, a large dietary PL load may result in greater amounts of intact PC reaching the distal parts of the intestine, where more bacteria reside and can contribute to the PC-TMA-TMAO pathway.

Daily egg intake has not been shown to be associated with CVD risk in healthy populations [11,12,13]. In contrast, several large cohort studies have found a positive association between egg intake and CVD in diabetics [11,12,101,102]. Consequently, patients with diabetes may be discouraged from eating eggs. These relationships are suggested to be caused by components found in the egg yolk, such as cholesterol and saturated fat [101]. It is also possible that the PC-TMA-TMAO pathway is more important or altered in diabetes. Serum TMAO levels have been shown to be elevated in those with type 2 diabetes [103]. This may be related to the kidney dysfunction that is often seen with diabetes, as TMAO levels can build up in the blood if they are not cleared by the kidneys. Regardless of kidney function, however, TMAO is a strong predictor of all-cause mortality risk [104,105]. If the plasma TMAO response to egg intake is increased in diabetics relative to non-diabetics, that may explain the consistent link between egg intake and CVD in diabetics. Further research is warranted to see if plasma TMAO response to egg intake is altered in insulin-resistant or diabetic humans.

## 8. Conclusions

**Figure 2:**
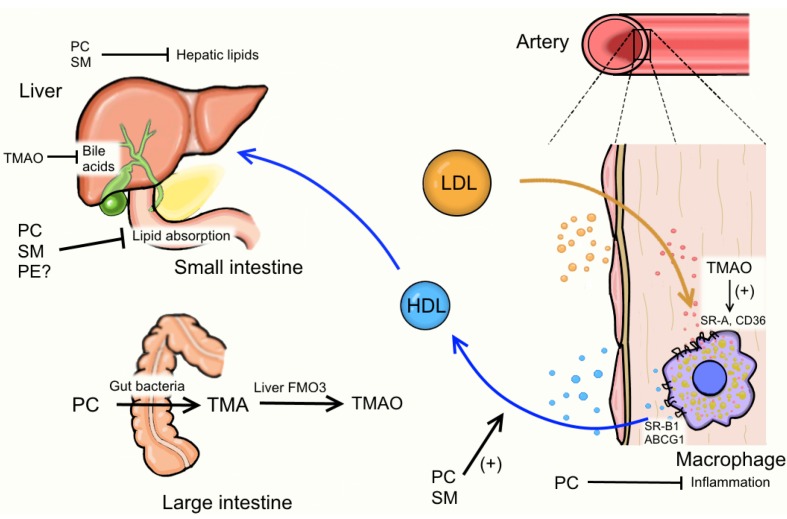
Potential pathways egg phospholipids could influence atherosclerosis. Egg phospholipids may lessen risk for cardiovascular disease (CVD) via reducing lipid absorption (PC, SM), reducing hepatic lipids (PC, SM), increasing HDL cholesterol efflux (PC, SM), and reducing inflammation (PC). Egg PC may also influence CVD risk via gut microflora-dependent catabolism to TMA and liver conversion to TMAO. TMAO may increase CVD risk via increasing macrophage scavenger receptors and decreasing RCT via reduced bile acid synthesis. Abbreviations: ABCG1, ATP-binding cassette transporter G1; CD36, Cluster of Differentiation 36; FMO3, flavin containing monooxygenase 3; HDL, high-density lipoprotein; LDL, low-density lipoprotein; PC, phosphatidylcholine; PE, phosphatidylethanolamine; SM, sphingomyelin; SR-A, scavenger receptor A; SR-B1, scavenger receptor B1; TMA, trimethylamine; TMAO, trimethylamine *N*-oxide.

Egg PL are important contributors to the overall dietary PL intake in the Western diet. Based on pre-clinical studies, egg PC and SM appear to regulate lipid absorption, hepatic lipid metabolism, and inflammation. In clinical studies, egg PL intake is associated with beneficial changes in serum biomarkers related to HDL function. However, the recent evidence linking acute ingestion of eggs with postprandial increases in plasma TMAO warrants concern. More research needs to be done to examine TMAO responses to chronic egg intake and in different populations, such as diabetics. It will be critical determine if the perceived benefits of egg PL intake on CVD risk markers outweigh the risk of potential TMAO formation (Figure 2).
